# Efficacy and safety of potassium-competitive acid blockers versus proton pump inhibitors as *Helicobacter pylori* eradication therapy: a meta-analysis of randomized clinical trials

**DOI:** 10.1016/j.clinsp.2022.100058

**Published:** 2022-07-08

**Authors:** Mengran Zhang, Mingge Pang, Mei Zhang

**Affiliations:** a Gastroenterology Department, Xuanwu Hospital Capital Medical University, Beijing, China; b Internal Medicine Department, Beijing Puren Hospital, Beijing, China

**Keywords:** Helicobacter pylori, Eradication therapy, Potassium-competitive acid blocker, Proton-pump inhibitor

## Abstract

**Background and aims:**

Potassium-Competitive Acid Blockers (P-CABs) have been used in *Helicobacter pylori* (*H. pylori*) eradication therapies in recent years. However, the efficacy and safety of P-CABs compared to Proton-Pump Inhibitors (PPIs) in this setting remain controversial.

**Methods:**

The efficacy and safety of P-CABs and PPIs for *H. pylori* eradication were compared in a meta-analysis based on a systematic literature search of major electronic databases for relevant Randomized Controlled Trials (RCTs).

**Results:**

Seven studies and 1,168 patients were included. The pooled eradication rate determined by Intention-To-Treat (ITT) analysis was 90.2% for P-CAB-based and 75.5% for PPI-based triple therapy (pooled RR [95% CI] = 1.17 [1.08–1.28], p < 0.001). The Per-Protocol (PP) analysis also demonstrated significant superiority of P-CABs (pooled eradication rate = 92.4% vs. 77.8%; pooled RR [95% CI] = 1.14 [1.03–1.26], p < 0.01). In a subgroup evaluation, P-CABs were significantly better than PPIs as a first-line eradication therapy, in both the ITT analysis (pooled eradication rate = 91.8% vs. 76.4%; pooled RR [95% CI] = 1.18 [1.10–1.28], p < 0.0001) and the PP analysis (pooled eradication rate = 93.0% vs. 78.6%; pooled RR [95% CI] = 1.13 [1.02–1.26], p < 0.05). However, P-CABs were not superior to PPIs when administered as salvage therapy, as determined in the ITT (75.0% vs. 66.0%, pooled RR [95% CI] = 1.11 [0.69–1.78], p = 0.66) and PP (85.7% vs. 70.0%, pooled RR [95% CI] = 1.20 [0.82–1.75], p = 0.34) analyses. In a subgroup analysis limited to Japanese patients, both the ITT analysis (pooled eradication rate = 89.6% vs. 73.9%; RR [95% CI] = 1.21 [1.14–1.29], p < 0.01) and the PP analysis (pooled eradication rate = 92.0% vs. 75.7%; RR [95% CI] = 1.18 [1.06–1.32], p < 0.01) showed that P-CABs were significantly superior compared to PPIs as triple eradication therapy. However, in the subgroup analysis of patients from other countries, there was no significant difference in either the ITT analysis (pooled eradication rate = 93.8% vs. 85.2%; RR [95% CI] = 1.10 [0.99–1.22], p = 0.07) or PP analysis (pooled eradication rate = 95.0% vs. 90.8%; RR [95% CI] = 1.05 [0.98–1.14], p = 0.17). The incidence of adverse events associated with the two regimens did not significantly differ (P-CABs vs. PPIs: 33.6% vs. 40.0%; RR [95% CI] = 0.84 [0.71‒1.00], p = 0.05). The incidence of serious adverse events and dropout rate due to adverse events also did not differ (p = 0.44 and p = 0.67, respectively).

**Conclusions:**

The efficacy of P-CAB-based triple therapy is superior to that of PPI-based triple therapy as a first-line approach to *H. pylori* eradication, particularly in Japanese patients. As salvage therapy, the efficacy of the two treatments did not significantly differ. The tolerability of P-CAB-based and PPI-based triple therapy was comparable, as was the incidence of adverse events.

## HIGHLIGHTS

• The efficacy of P-CAB-based triple therapy is superior to that of PPI-based triple therapy as a first-line approach to *H. pylori* eradication, particularly in Japanese patients.
• P-CABs were not superior to PPIs as a salvage triple eradication therapy.
• The safety and tolerability of P-CAB are comparable to PPI in *H. pylori* triple eradication therapies.
• Further large RCTs conducted in multiple regions and countries are necessary.

## Introduction

*Helicobacter pylori* (*H. pylori*) infection is one of the most common infectious diseases worldwide, affecting approximately half of the global population^1^ and playing a causative role in a number of gastrointestinal diseases, including asymptomatic chronic gastritis, peptic ulcer disease, atrophic gastritis, intestinal metaplasia, gastric mucosa-associated lymphoid tissue lymphoma, and gastric adenocarcinoma.^2^, ^3^, ^4^ Both the Maastricht V Consensus Report and Kyoto Global Consensus Report recognize *H. pylori*-associated gastritis as an infectious disease, while the World Health Organization has designated *H. pylori* as a carcinogen. There is also accumulating evidence that *H. pylori* eradication can reduce the incidence of gastric cancer.^5^, ^6^, ^7^, ^8^

Proton-Pump Inhibitor (PPI) regimens are the most common first-line and salvage therapies for *H. pylori* eradication. However, in recent years, the success rate of PPI-containing regimens has declined, due to the increased antibiotic resistance of *H. pylori*.^9^^,^^10^ A combination of effective antibiotics and acid suppression is needed for successful eradication, but the optimal treatment regimen has yet to be determined.

Potassium-Competitive Acid Blockers (P-CABs) inhibit gastric acid secretion via selective and reversible inhibition of H+/K+-ATPase. Since P-CABs compete with K+, their activity is dose-dependent. Moreover, the onset of P-CAB activity is faster than that of PPI activity; moreover, acid suppression by P-CABs is of longer duration, making the latter group of drugs more potent therapies for *H. pylori* eradication.^11^^,^^12^ Furthermore, because P-CABs are acid-stable and less impaired by the CYP2C19 system than PPIs,^13^^,^^14^ their use in anti-*H. pylori* regimens have garnered considerable interest.^14^^,^^15^

P-CABs are currently mainly approved in Asia. Vonoprazan (VPZ) is a first-in-class P-CAB available in Japan since 2015, which has also been introduced in a small number of other Asian countries,^16^, ^17^, ^18^, ^19^ mainly for gastroesophageal reflux disease and *H. pylori* treatment. Other P-CABs include revaprazan, tegoprazan, linaprazan, YH4808, DWP14012, KFP-H008, and SCH28080. Several recent meta-analyses have shown the superiority of P-CAB-containing therapies over PPI-containing therapies,^20^, ^21^, ^22^, ^23^ although they mostly included retrospective studies with low levels of evidence, which limited the accuracy and reliability of the results. In addition, the conclusions were inconsistent with those of other studies.^20^^,^^24^, ^25^, ^26^ Therefore, the authors performed a meta-analysis that included only Randomized Controlled Trials (RCTs) to assess the efficacy and safety of P-CAB-based therapy for *H. pylori* eradication.

## Methods

### Search strategy

The PubMed, Embase, and Cochrane Library databases were systematically searched for relevant RCTs up to November 10, 2021. The following search string was used: (“potassium-competitive acid blocker” or “vonoprazan” or “takecab” or “TAK438”) and (“Helicobacter pylori” or “*H. pylori*” or “*Hp*”). Related articles and citations were also considered to broaden the search. All human studies published in English were initially included. The search was conducted by two independent reviewers (Zhang Mengran and Zhang Mei); a third author (Pang Mingge) was consulted whenever disagreements arose.

### Study selection

Two reviewers (ZMR and ZM) independently reviewed the full-text versions of all articles retrieved in the literature search to identify eligible studies. The inclusion criteria were as follows: clinical RCT comparing P-CAB- and PPI-based therapy as the primary or salvage regimen for *H. pylori* eradication; *H. pylori* infection confirmed (with one or more confirmatory tests) by a Urea Breath Test (UBT), rapid urease test, culture, or stool *H. pylori* antigen; eradication rate assessed by Intention-To-Treat (ITT) and Per-Protocol (PP) analyses at least 4-weeks after the completion of treatment; and confirmation of *H. pylori* eradication, either by UBT or stool antigen test.

The exclusion criteria were as follows: non-RCT; inadequate or unavailable data; abstract-only publications or unpublished; language other than English; and eradication rate not assessed.

### Study quality assessment

Two reviewers (ZMR and ZM) independently assessed the risk of bias of included RCTs using the Cochrane Risk of Bias assessment tool. The following items of each RCT were evaluated: (i) Methods of random allocation; (ii) How patient allocation was concealed; (iii) Blinding of the patients and researchers; (iv) Blinding of outcome assessment; (v) Whether there were incomplete outcome data; (vi) Whether there was selective outcome reporting; and (vii) Other potential biases.

### Data extraction

Data extraction was undertaken by two investigators, independently. For each eligible study, the following data were extracted: first author, year of publication, study design, country, study period, eradication regimens, confirmative test for eradication, eradication rate, dropout rate, and adverse events.

### Statistical analysis

Meta-analysis was performed to calculate the pooled Risk Ratios (RRs) with a 95% Confidence Interval (CI) by using Review Manager 5.3 (provided by the Cochrane Collaboration, 2014). The Chi-Square test and Higgins I^2^ statistic were used to estimate the heterogeneity between different studies. When p > 0.1 and I^2^ < 50%, it was considered that there was no heterogeneity existed, and the fixed-effect model was used. Otherwise, heterogeneity was considered to exist, and subgroup analysis or a randomized effect model was used. All p-values were two-tailed, and p < 0.05 were considered statistically significant in all tests (except for the heterogeneity test).

## Results

### Study selection and characteristics

**Figure 1:**
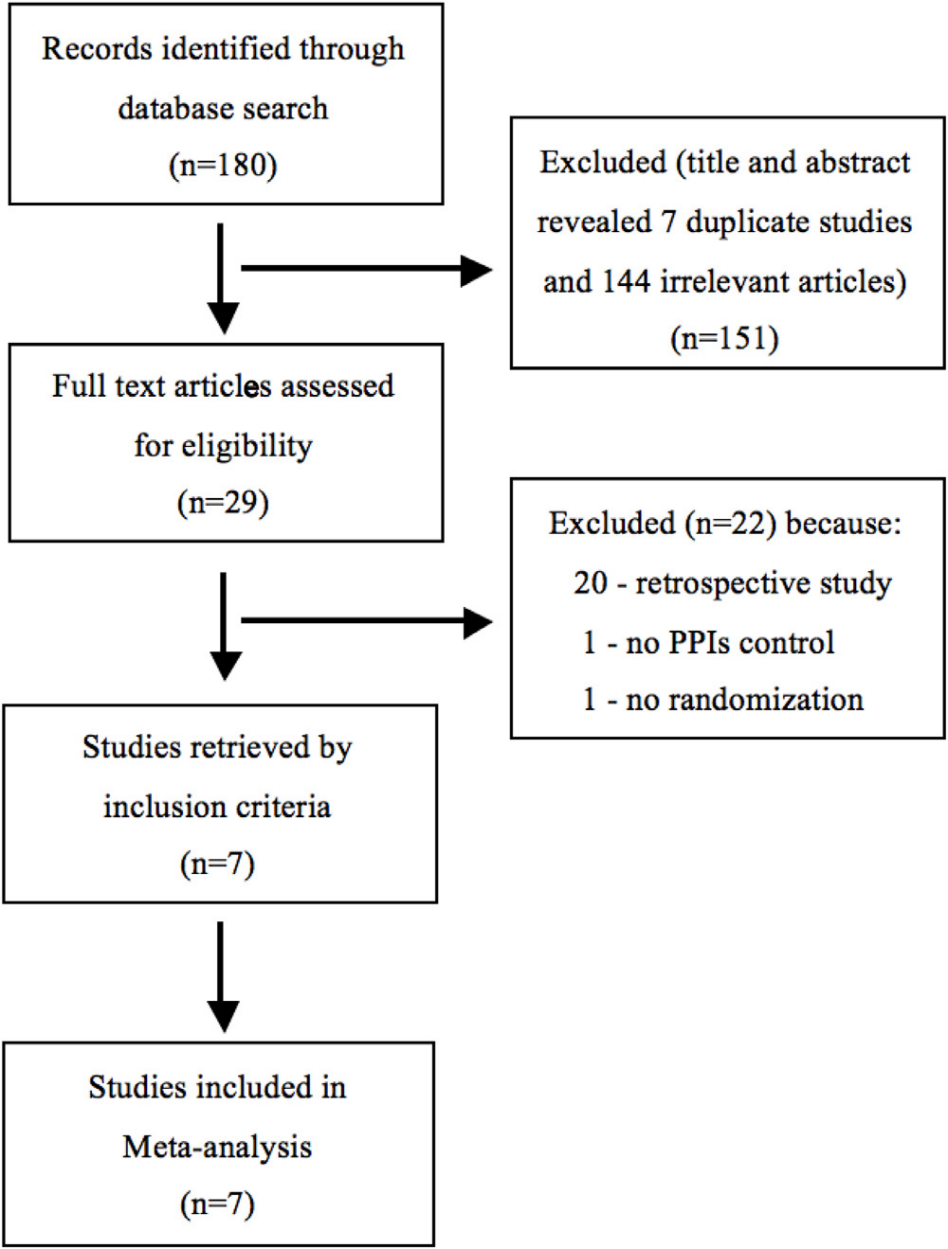
Flowchart for study selection.

Figure 1 shows a flow diagram of the present literature search. Initially, 180 studies were identified. After screening the titles and abstracts, 7 duplicate and 144 irrelevant articles were discarded. After reviewing the full-length articles, 22 were excluded based on the exclusion criteria. Ultimately, seven studies, comprising 1,168 patients and meeting all eligibility criteria, were included in the present meta-analysis.^24^, ^25^, ^26^, ^27^, ^28^, ^29^, ^30^

The characteristics of the seven studies are summarized in Table 1. The studies were published between 2016 and 2021, and their enrollment periods ranged from 2012 to 2021. All of the included studies were performed in Asia, including one study conducted in Korea and one in Thailand; the others were all conducted in Japan.

**Table 1:** Characteristics of studies included in the meta-analysis.

First author	Year of publication	Country	Study period	Dosage of P-CAB	Dosage of antibiotics and PPI
Bunchorntavakul^24^	2021	Thailand	2019‒2021	VPZ 20 mg, bd, 7 days	A1000 mg bd, C 500 mg
				OPZ 20 mg, bd, 14 days	bd, 7 days or 14 days
Hojo^25^	2020	Japan	2015‒2017	VPZ 20 mg, bd, 7 days	A 750 mg bd, M 250 mg
				RPZ 10 mg, bd, 7 days	bd, 7 days
Park^26^	2020	Korea	2013	YH4808 200 mg, bd, 7 days	A1000 mg bd, C 500 mg
				ESO 20 mg, bd, 7 days	bd, 7 days
Murakami^27^	2016	Japan	2012‒2013	VPZ 20 mg, bd, 7 days	A 750 mg bd, C
				LPZ 30 mg, bd, 7 days	200 or 400 mg, bd, 7 days
Maruyama^28^	2017	Japan	2015‒2016	VPZ 20 mg, bd, 7 days	A 750 mg bd, C 200
				RPZ 20 mg or LPZ 30 mg	or 400 mg, bd, 7 days
				bd, 7 days	
Sue^29^	2019	Japan	2015‒2017	VPZ 20 mg, bd, 7 days	A 750 mg bd, S 100 mg
				ESO 20 mg, RPZ 10 mg or bd, 7 days	LPZ 30 mg, bd, 7 days
Sue^30^	2018	Japan	2015‒2016	VPZ 20 mg, bd, 7 days	A 750 mg bd, C 200
				ESO 20 mg, RPZ 10 mg or 400 mg, bd, 7 days	LPZ 30 mg, bd, 7 days

VPZ, Vonoprazan; OPZ, Omeprazole; RPZ, Rabeprazole; ESO, Esomeprazole; LPZ, Lansoprazole; A, Amoxicillin; C, Clarithromycin; M, Metronidazole; S, Sitafloxcin.

Among the 1,168 patients included in this meta-analysis, 593 received P-CAB-based eradication therapy (YH4804 administered to 20 patients in one study; the remainder received VPZ) and 575 patients received PPI-based therapy. The 1,059 patients in five studies were treatment-naïve; in the other studies, primary treatment had failed. P-CAB-based triple therapy, as first-line therapy, consisted of 20 mg VPZ or 200 mg YH4804, 750 or 1,000 mg amoxicillin, and 200 or 400 mg clarithromycin, twice daily for 7-days. P-CAB-based triple salvage therapy consisted of 20 mg VPZ, 750 mg amoxicillin, and 250 mg metronidazole or 100 mg sitafloxacin, twice daily for 7-days. In PPI-based triple first-line or salvage therapy, a standard dose of a PPI (20 mg omeprazole, 20 mg esomeprazole, 30 mg lansoprazole, or 10 or 20 mg rabeprazole) was administered. In all seven studies, eradication success was determined based on a UBT conducted at least 4-weeks after treatment completion.

### Risk of bias

**Figure 2:**
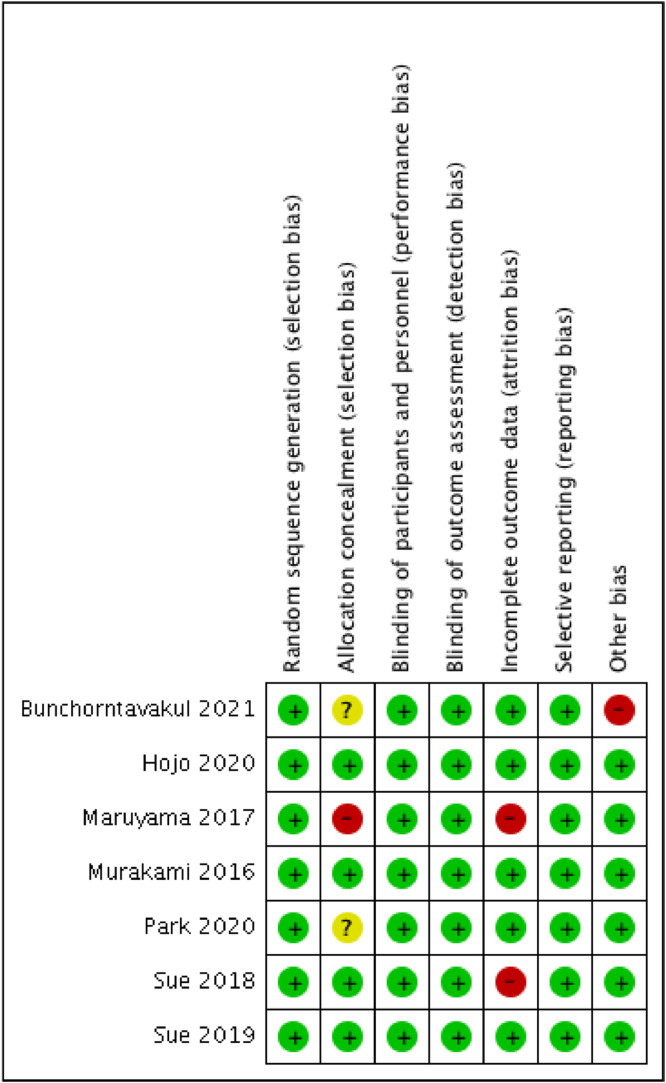
Assessment of bias risk.

The risk of bias was assessed using the Cochrane Collaboration tool; the results are presented in Figure 2. Generally, the included studies had a low risk of bias, but three studies had a high risk of bias.

### Comparative eradication success rate

**Figure 3:**
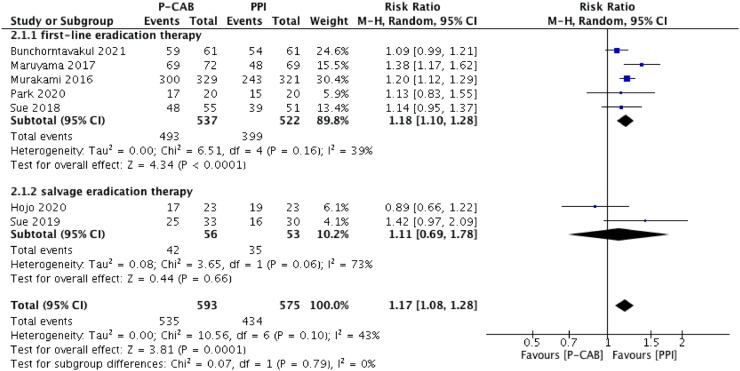
Forest plot of P-CABs versus PPIs for *H. pylori* eradication rate in intention-to-treat analysis.

**Figure 4:**
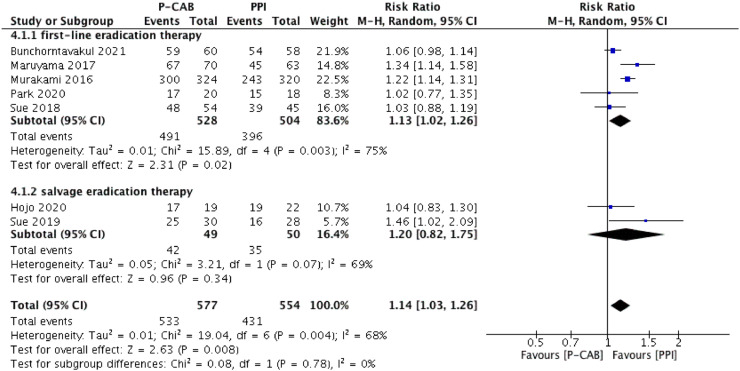
Forest plot of P-CABs versus PPIs for *H. pylori* eradication rate in per-protocol analysis.

The pooled eradication rate determined by ITT analysis was 90.2% for P-CAB-based triple therapy and 75.5% for PPI-based triple therapy. As shown in Figure 3, a higher *H. pylori* eradication rate was achieved with P-CAB-based triple therapy than with PPI-based triple therapy (pooled RR [95% CI] = 1.17 [1.08–1.28], p < 0.001). No significant heterogeneity was identified (I^2^ = 43%). A similar tendency was determined in the PP analysis (pooled eradication rate = 92.4% vs. 77.8%; pooled RR [95% CI] = 1.14 [1.03–1.26], p < 0.01), as shown in Figure 4, but significant heterogeneity was identified (I^2^ = 68%).

### Subgroup analysis of patients receiving primary (first-line) or salvage (second- or third-line) therapy

A subgroup analysis was performed according to therapy type (first-line or salvage eradication therapy); (Figs. 3 and 4). In the first-line therapy subgroup, the eradication efficacy of P-CAB-based triple therapy was superior to that of PPI-based triple therapy, according to both the ITT analysis (pooled eradication rate = 91.8% vs. 76.4%; pooled RR [95% CI] = 1.18 [1.10–1.28], p < 0.0001) and the PP analysis (pooled eradication rate = 93.0% vs. 78.6%; pooled RR [95% CI] = 1.13 [1.02–1.26], p < 0.05). Significant heterogeneity was identified in the PP analysis (I^2^ = 75%) but not in the ITT analysis (I^2^ = 39%). Among patients who received P-CAB-based and PPI-based salvage therapy, the pooled eradication rate, as determined by ITT analysis, was 75.0% and 66.0%, respectively (pooled RR [95% CI] = 1.11 [0.69–1.78], p = 0.66) whereas in the PP analysis it was 85.7% and 70.0% (pooled RR [95% CI] = 1.20 [0.82–1.75], p = 0.34). Although P-CAB-based salvage therapy tended to be superior to PPI-based salvage therapy with respect to eradication efficacy, the difference was not statistically significant in either the ITT or PP analysis, although in both analyses the heterogeneity was significant (I^2^ = 73% and 69%, respectively).

### Subgroup analysis according to country

**Figure 5:**
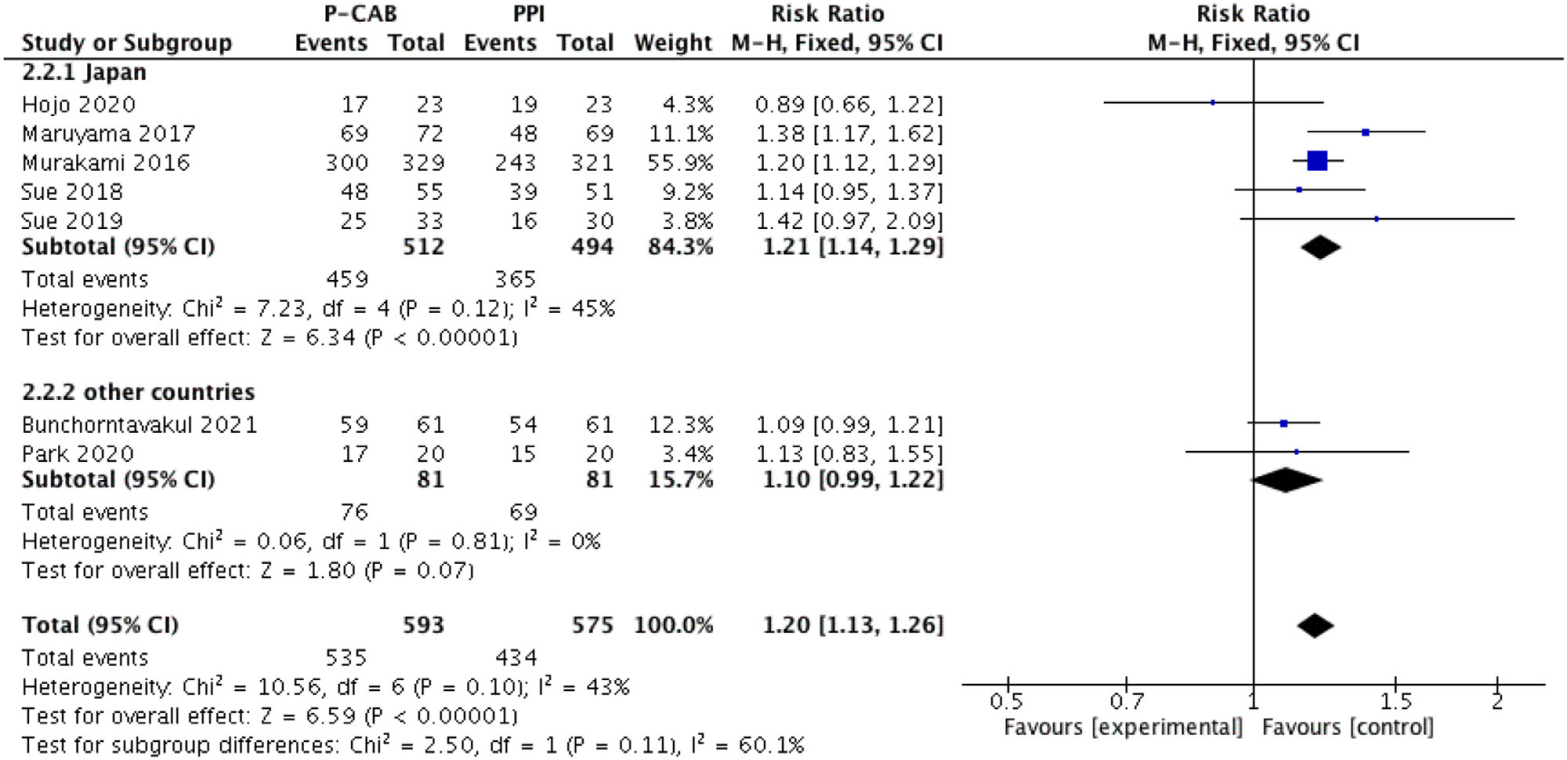
Forest plot of P-CABs versus PPIs for *H. pylori* eradication rate in Japan/other countries subgroup according to intention-to-treat analysis.

**Figure 6:**
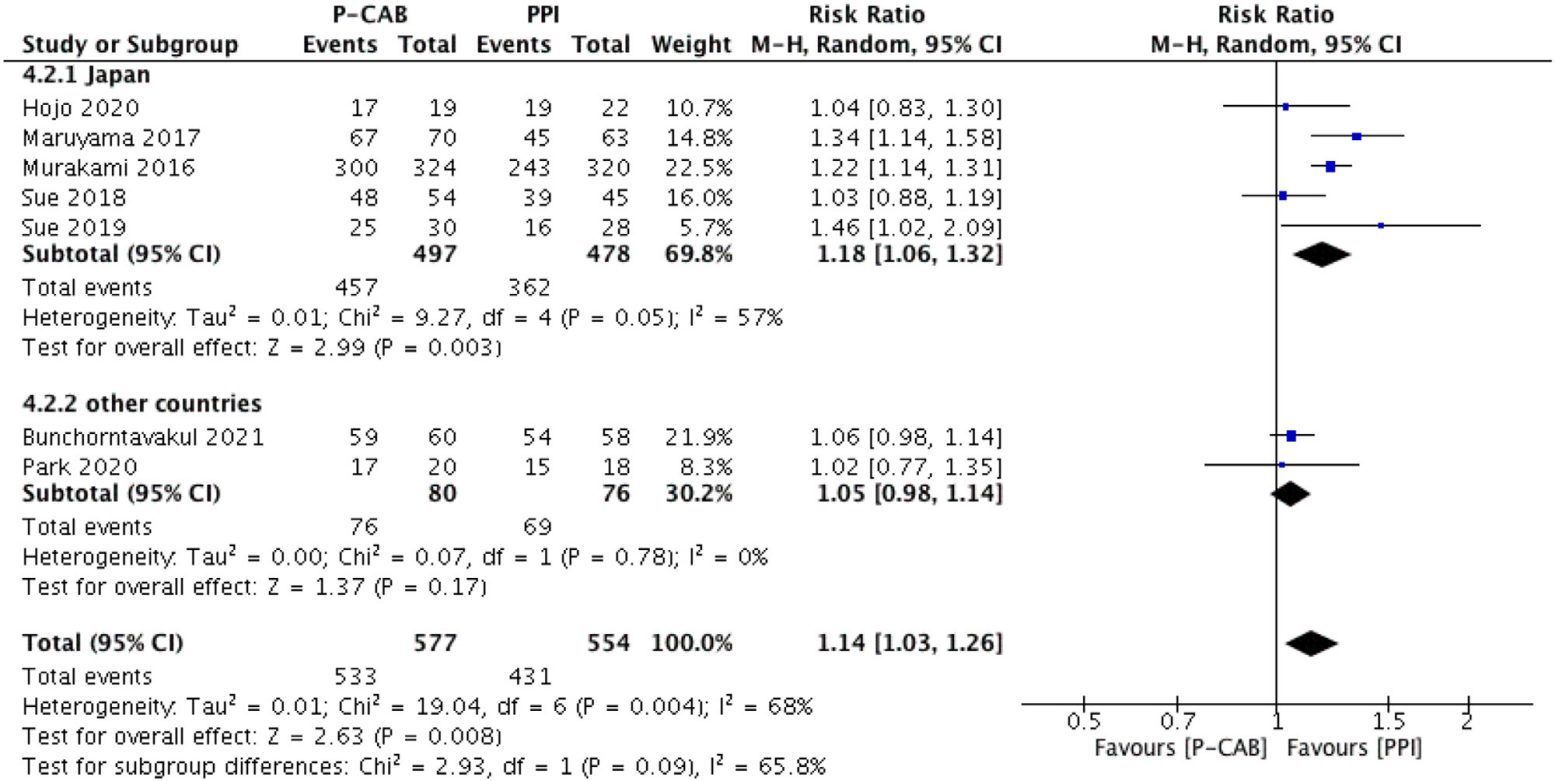
Forest plot of P-CABs versus PPIs for *H. pylori* eradication rate in Japan/other countries subgroup according to per-protocol analysis.

A subgroup analysis was performed according to whether the study was conducted in Japan or in other countries (Figure 5, Figure 6). In the subgroup of Japanese studies, the forest plot analysis showed significant superiority of P-CAB- over PPI-based regimens in terms of the overall success of *H. pylori* eradication, in both the ITT analysis (pooled eradication rate = 89.6% vs. 73.9%; RR [95% CI] = 1.21 [1.14–1.29], p < 0.01) and PP analysis (pooled eradication rate = 92.0% vs. 75.7%; RR [95% CI] = 1.18 [1.06–1.32], p < 0.01). Significant heterogeneity was identified in the PP analysis (I^2^ = 57%) but not in the ITT analysis (I^2^ = 45%). However, in the subgroup consisting of studies from other countries, the eradication efficacy of P-CAB-based therapy was not superior to that of PPI-based therapy, in either the ITT analysis (pooled eradication rate = 93.8% vs. 85.2%; RR [95% CI] = 1.10 [0.99–1.22], p = 0.07) or PP analysis (pooled eradication rate = 95.0% vs. 90.8%; RR [95% CI] = 1.05 [0.98–1.14], p = 0.17). There was also no significant heterogeneity (I^2^ = 0%).

### Adverse events

**Figure 7:**
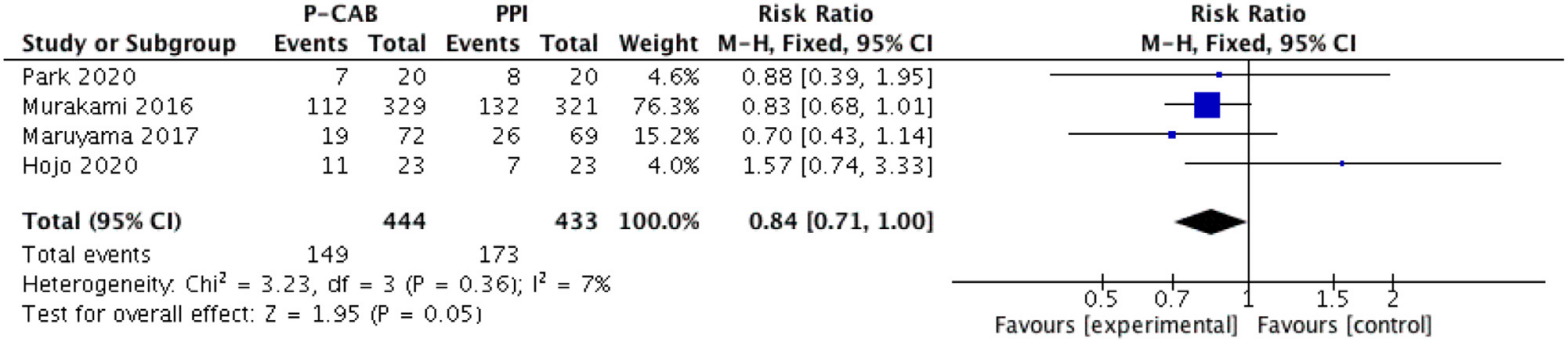
Forest plot of adverse events between P-CABs versus PPIs.

All included studies provided detailed information regarding adverse events, but only four reported the overall incidence thereof, which was 33.6% for P-CAB-based therapy and 40.0% for PPI-based therapy. There was no difference in the rate of adverse events between the two regimens (RR [95% CI] = 0.84 [0.71–1.00], p = 0.05), nor was there significant heterogeneity (I^2^ = 7%), as shown in Figure 7. Among the included studies, four reported that adverse events caused seven and four patients receiving P-CAB- and PPI-based regimens to stop treatment, respectively. One of the four studies reported that four serious adverse events occurred in patients receiving P-CAB-based therapy, and two in those receiving PPI-based therapy. In the other three studies, no patient experienced a serious adverse event or side effect leading to treatment discontinuation. The overall incidence of serious adverse events was 0.67% among patients in the P-CAB-based therapy group and 0.35% among those in the PPI-based therapy group; the difference was not significant (p = 0.44); (Fig. S1). The pooled dropout rate due to adverse events was 1.2% in P-CAB-treated patients and 0.7% in PPI-treated patients. There was no significant difference in the dropout rate between the two regimens, nor was there significant heterogeneity (RR [95% CI] = 1.53 [0.51‒4.63], p = 0.67, I^2^ = 0%); (Fig. S2). The most common adverse events were diarrhea, dysgeusia, abdominal fullness, abdominal pain, anorexia, nausea, headache, and belching.

## Discussion

The current regimen approved for *H. pylori* eradication combines a PPI and two antibiotics, with or without bismuth. However, the success of this regimen has been limited, mostly due to antibiotic resistance and insufficient gastric acid suppression. The advantages of a P-CAB-containing eradication regimen include rapid onset of action, long-duration acid suppression, less interindividual variation in acid suppression, and a minimal influence of diet on its action. Thus, with P-CAB-based therapies, the eradication rate is expected to be higher than that achieved by conventional regimens using a standard dose of a PPI. To date, the most commonly used P-CAB has been VPZ, administered in Japanese populations, although other P-CABs have been tested in other Asian countries.

The present study's meta-analysis revealed a higher eradication rate with P-CAB- than with PPI-based triple therapy, as determined in an ITT analysis (89.0% vs. 74.2%) and PP analysis (89.0% vs. 74.2%). These results are consistent with a previous meta-analysis that reported eradication rates of 87.9% for VPZ-based therapy and 72.8% for PPI-based therapy.^21^ The superiority of VPZ was demonstrated in a subgroup analysis of treatment-naïve patients. The results showed that this P-CAB was superior to PPIs when administered to treatment-naive patients as first-line triple therapy (eradication rate = 91.8% vs. 76.4% in the ITT analysis and 93.0% vs. 78.6% in the PP analysis). Specifically, the eradication rate of P-CAB-containing first-line therapy was > 90%, which implies high efficacy and accords with the recommendations of currently available guidelines. By contrast, the eradication rate of PPI-based therapy is < 80%, which is considered unacceptable. The potent acid-inhibitory effect of P-CABs may explain its high efficacy. In a previous meta-analysis, high-dose PPIs were shown to be more effective than standard-dose PPPIs for eradicating *H. pylori* infection,^31^ because the increased gastric pH may drive *H. pylori* to re-enter the replicative state and thus become susceptible to antibiotics.^32^^,^^33^ However, in salvage therapy, clarithromycin is replaced by metronidazole or sitafloxacin. With this regimen, there was no significant difference in eradication rate between P-CAB-containing and PPI-containing triple therapy, either in the ITT analysis (75.0% vs. 66.0%, p = 0.66) or PP analysis (85.7% vs. 70.0%, p = 0.34). These findings are consistent with a previous study,^20^ in which the efficacy of P-CABs (VPZ) was superior to that of PPIs as first-line *H. pylori* eradication therapy in patients infected with either clarithromycin-susceptible or clarithromycin-resistant strains. However, VPZ was not superior to PPIs as second-line triple eradication therapy. This lack of a difference may be due to the fact that the efficacy of acid inhibitors is an important factor in the context of clarithromycin therapy, but not metronidazole or sitafloxacin therapy. Contrary findings were presented in a previous meta-analysis of retrospective studies,^23^ in which VPZ-based regimens were shown to be significantly superior to PPI-based regimens as second-line *H. pylori* eradication therapy. In the present study's salvage therapy subgroup, the small number and significant heterogeneity of the included studies limited the level of evidence.

P-CABs for *H. pylori* eradication is mainly used in Japan, such that most studies included Japanese patients. Therefore, to examine the efficacy of P-CABs in patients from other countries, the authors conducted another subgroup analysis according to country. In the subgroup of patients from Japan, a higher *H. pylori* eradication rate was obtained with the P-CAB- than the PPI-based regimen, in both the ITT analysis (89.6% vs. 93.9%) and PP analysis (92.0% vs. 75.7%). However, this was not the case in the subgroup of patients from countries other than Japan, as the difference between the two treatment groups was not significant either in the ITT analysis (p = 0.07) or PP analysis (p = 0.17). However, because the two studies in this subgroup had small sample sizes, the results need to be interpreted with caution.

In the present meta-analysis, the safety of P-CABs and PPIs as *H. pylori* eradication therapies was also assessed. There was no significant difference between the two groups in terms of the incidence of adverse or serious adverse events. Nonetheless, the dropout rate related to adverse events was lower in the P-CAB than the PPI group. Therefore, the safety and tolerance of P-CAB-containing *H. pylori* eradication therapies are acceptable.

Although the present study's meta-analysis demonstrated the benefit of P-CAB-based eradication therapy, it had several limitations. First, all of the included studies were performed in Asian countries; the lack of data from European and American countries may have led to selection bias, given that regional differences in diets and genetics influence gastric pH. Second, the number of studies and sample sizes used to analyze the difference between P-CABs and PPIs as salvage therapy, and in countries other than Japan, was limited. Third, the role of antibiotic (e.g., clarithromycin) resistance was not assessed. Fourth, different antibiotics were used for salvage therapy. Lastly, only conventional triple therapies provided for 7-days were compared in the meta-analysis; alternative therapeutic strategies were not considered. Large RCTs conducted in multiple regions and countries are necessary to confirm the authors’ findings.

## Conclusion

In summary, a comparison of the efficacy of first-line *H. pylori* triple eradication therapies showed that P-CABs were superior to PPI. The present meta-analysis also highlighted that the superiority of P-CABs was mainly driven by studies conducted in Japan. However, P-CABs were not superior to PPIs as a salvage triple eradication therapy. In addition, the present study showed that the safety of P-CABs for *H. pylori* eradication is comparable to PPIs. Nevertheless, as some of the authors’ conclusions were based on small samples with significant heterogeneity, they should be interpreted with caution. Further large-scale RCTs are needed to validate the present findings.

## Authors’ contributions

Zhang Mengran: Concept, design, literature search, data collection and/or processing; analysis and/or interpretation; writing manuscript.

Pang Mingge: Literature search, data collection and/or processing.

Zhang Mei: Concept, design, supervision, resources, critical review.

## Conflicts of interest

The authors declare no conflicts of interest.

## Appendix Supplementary materials

Image, application 1

## References

[1] Hooi JKY, Lai WY, Ng WK, Suen MMY, Underwood FE, Tanyingoh D (2017). Global prevalence of *Helicobacter pylori* infection: systematic review and meta-analysis. Gastroenterology.

[2] O'Connor A, O'Morain CA, Ford AC. (2017). Population screening and treatment of *Helicobacter pylori* infection. Nat Rev Gastroenterol Hepatol.

[3] Malfertheiner P, Chan FK, McColl KE. (2009). Peptic ulcer disease. Lancet.

[4] Fock KM, Graham DY, Malfertheiner P. (2013). *Helicobacter pylori* research: historical insights and future directions. Nat Rev Gastroenterol Hepatol.

[5] Graham DY. (2015). *Helicobacter pylori* update: gastric cancer, reliable therapy, and possible benefits. Gastroenterology.

[6] Malfertheiner P, Link A, Selgrad M. (2014). *Helicobacter pylori*: perspectives and time trends. Nat Rev Gastroenterol Hepatol.

[7] Lee YC, Lin JT. (2017). Screening and treating *Helicobacter pylori* infection for gastric cancer prevention on the population level. J Gastroenterol Hepatol.

[8] Sugano K. (2019). Effect of *Helicobacter pylori* eradication on the incidence of gastric cancer: a systematic review and meta-analysis. Gastric Cancer.

[9] Lee JY, Park KS. (2016). Optimal first-line treatment for *Helicobacter pylori* infection: recent strategies. Gastroenterol Res Pract.

[10] Kuo CH, Kuo FC, Hu HM, Liu CJ, Wang SS, Chen YH (2012). The optimal first-line therapy of *Helicobacter pylori* infection in year 2012. Gastroenterol Res Pract.

[11] Scott DR, Munson KB, Marcus EA, Lambrecht NW, Sachs G. (2015). The binding selectivity of vonoprazan (TAK-438) to the gastric H+, K+ -ATPase. Aliment Pharmacol Ther.

[12] Park JY, Kim JG. (2018). New *Helicobacter pylori* eradication therapies. Korean J Gastroenterol.

[13] Guevara B, Cogdill AG. (2020). *Helicobacter pylori*: a review of current diagnostic and management strategies. Dig. Dis. Sci..

[14] Graham DY, Dore MP. (2018). Update on the use of vonoprazan: a competitive acid blocker. Gastroenterology.

[15] Lyu QJ, Pu QH, Zhong XF, Zhang J. (2019). Efficacy and safety of vonoprazan-based versus proton pump inhibitor-based triple therapy for *Helicobacter pylori* eradication: a meta-analysis of randomized clinical trials. Biomed Res Int.

[16] Garnock-Jones KP (2015). Vonoprazan: first global approval. Drugs.

[17] Echizen H. (2016). The first-in-class potassium-competitive acid blocker, vonoprazan fumarate: pharmacokinetic and pharmacodynamic considerations. Clin Pharmacokinet.

[18] Hunt RH, Scarpignato C. (2018). Potent acid suppression with PPIs and P-CABs: what's new?. Curr Treat Options Gastroenterol.

[19] Scarpignato C, Hunt RH. (2019). The potential role of potassium-competitive acid blockers in the treatment of gastroesophageal reflux disease. Curr Opin Gastroenterol.

[20] Dong SQ, Singh TP, Wei X, Yao H, Wang HL. (2017). Review: A Japanese population-based meta-analysis of vonoprazan versus PPI for *Helicobacter pylori* eradication therapy: Is superiority an illusion?. Helicobacter.

[21] Jung YS, Kim EH, Park CH. (2017). Systematic review with meta-analysis: the efficacy of vonoprazan-based triple therapy on *Helicobacter pylori* eradication. Aliment Pharmacol Ther.

[22] Li M, Oshima T, Horikawa T, Tozawa K, Tomita T, Fukui H (2018). Systematic review with meta-analysis: vonoprazan, a potent acid blocker, is superior to proton-pump inhibitors for eradication of clarithromycin-resistant strains of *Helicobacter pylori*. Helicobacter..

[23] Shinozaki S, Kobayashi Y, Osawa H, Sakamoto H, Hayashi Y, Lefor AK (2021). Effectiveness and safety of vonoprazan versus proton pump inhibitors for second-line *Helicobacter pylori* eradication therapy: systematic review and meta-analysis. Digestion.

[24] Bunchorntavakul C, Buranathawornsom A. (2021). Randomized clinical trial: 7-day vonoprazan-based versus 14-day omeprazole-based triple therapy for *Helicobacter pylori*. J Gastroenterol Hepatol.

[25] Hojo M, Asaoka D, Takeda T, Shimada Y, Matsumoto K, Matsumoto K (2020). Randomized controlled study on the effects of triple therapy including vonoprazan or rabeprazole for the second-line treatment of *Helicobacter pylori* infection. Therap Adv Gastroenterol.

[26] Park H, Kim CO, Kim M, Lim Y, Lee WY, Yoon S (2020). Pharmacodynamic evaluation of YH4808 for *Helicobacter pylori* eradication in healthy subjects. Transl Clin Pharmacol.

[27] Murakami K, Sakurai Y, Shiino M, Funao N, Nishimura A, Asaka M (2016). Vonoprazan, a novel potassium-competitive acid blocker, as a component of first-line and second-line triple therapy for *Helicobacter pylori* eradication: a phase III, randomised, double-blind study. Gut.

[28] Maruyama M, Tanaka N, Kubota D, Miyajima M, Kimura T, Tokutake K (2017). Vonoprazan-based regimen is more useful than PPI-based one as a first-line *Helicobacter pylori* eradication: a randomized controlled trial. Can J Gastroenterol Hepatol.

[29] Sue S, Shibata W, Sasaki T, Kaneko H, Irie K, Kondo M (2019). Randomized trial of vonoprazan-based versus proton-pump inhibitor-based third-line triple therapy with sitafloxacin for *Helicobacter pylori*. J Gastroenterol Hepatol.

[30] Sue S, Ogushi M, Arima I, Kuwashima H, Nakao S, Naito M (2018). Vonoprazan- vs proton-pump inhibitor-based first-line 7-day triple therapy for clarithromycin-susceptible *Helicobacter pylori*: A multicenter, prospective, randomized trial. Helicobacter..

[31] Villoria A, Garcia P, Calvet X, Gisbert JP, Vergara M. (2008). Meta-analysis: high-dose proton pump inhibitors vs. standard dose in triple therapy for *Helicobacter pylori* eradication. Aliment Pharmacol Ther.

[32] Graham DY, Fischbach L. (2010). *Helicobacter pylori* treatment in the era of increasing antibiotic resistance. Gut.

[33] Graham DY, Shiotani A. (2008). New concepts of resistance in the treatment of *Helicobacter pylori* infections. Nat Clin Pract Gastroenterol Hepatol.

