# Supplementary material for: Efficacy and safety of potassium-competitive acid blockers versus proton pump inhibitors as Helicobacter pylori eradication therapy: a meta-analysis of randomized clinical trials
Source: Clinics (Sao Paulo). 2022 Jul 8;77:100058. doi: 10.1016/j.clinsp.2022.100058 (PMC9278030; doi:10.1016/j.clinsp.2022.100058)

CLINICS-D-22-00047 – Supplementary Material

**Figure S1** Forest plot of serious adverse events between P-CABs versus PPIs.


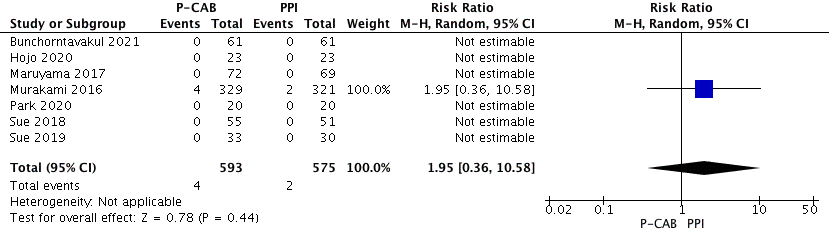


**Figure S2** Forest plot of dropout rate between P-CABs versus PPIs.


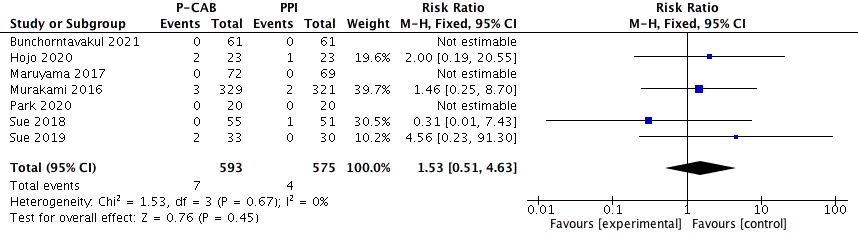

Supplement: Supplementary file 1 [file mmc1.docx]
